# Improved Early Detection Models of Pharyngocutaneous Fistula after Total Laryngectomy

**DOI:** 10.3390/jcm12051851

**Published:** 2023-02-26

**Authors:** Yujin Heo, Hyun Suk Lee, Sungha Jung, Changhee Lee, Younghac Kim, Man Ki Chung, Han-Sin Jeong, Chung-Hwan Baek, Joong Hyun Ahn, Young-Ik Son, Nayeon Choi

**Affiliations:** 1 Department of Otorhinolaryngology, Head and Neck Surgery, Samsung Medical Center, Sungkyunkwan University School of Medicine, Seoul 06351, Republic of Korea; 2 Department of Internal Medicine-Nephrology, Samsung Medical Center, Sungkyunkwan University School of Medicine, Seoul 06351, Republic of Korea; 3 Biomedical Statistics Center, Institute of Future Medicine, Samsung Medical Center, Sungkyunkwan University School of Medicine, Seoul 06351, Republic of Korea

**Keywords:** total laryngectomy, pharyngocutaneous fistula, early detection, method, fistulography

## Abstract

Early detection of pharyngocutaneous fistula (PCF) after total laryngectomy (TL) could prevent severe complications such as major vessel rupture. We aimed to develop prediction models for detecting PCF in the early postoperative period. We retrospectively analyzed patients (*N* = 263) who received TL between 2004 and 2021. We collected clinical data for fever (>38.0 °C) and blood tests (WBC, CRP, albumin, Hb, neutrophils, lymphocytes) on postoperative days (POD) 3 and 7, and fistulography on POD 7. Clinical data were compared between fistula and no fistula groups, and significant factors were selected using machine learning. Using these clinical factors, we developed improved prediction models for PCF detection. Fistula occurred in 86 (32.7%) patients. Fever was significantly (*p* < 0.001) more common in the fistula group, and ratios (POD 7 to 3) of WBC, CRP, neutrophils, and neutrophils-to-lymphocytes (NLR) were significantly higher (all *p* ≤ 0.001) in the fistula group than in the no fistula group. Leakage on fistulography was more common in the fistula group (38.2%) than in the no fistula group (3.0%). The area under curve (AUC) of fistulography alone was 0.68, but predictive models using a combination of fistulography, WBC at POD 7, and neutrophil ratio (POD 7/POD 3) showed better diagnostic performance (AUC of 0.83). Our predictive models may detect PCF early and accurately, which could reduce fatal complications following PCF.

## 1. Introduction

Total laryngectomy (TL) is one of the treatment options for advanced laryngeal and hypopharyngeal cancers, especially in salvage cases. Pharyngocutaneous fistula (PCF) is the most common and devastating complications of TL and can result in major vessel rupture, delayed adjuvant therapy, increased hospitalization duration, higher medical expenses, and reduced quality of life [1,2,3]. The incidence of PCF after TL is 3.3% to 65% [4,5,6]. PCF usually occurs within two weeks after TL [7]. Although most patients with PCF can be managed conservatively, some require surgical intervention because of prolonged fistula, surgical wound infection, or impending rupture of major vessels [8,9]. Therefore, early detection and proper management of PCF are crucial for preventing critical complications. In addition, early detection of PCF could minimize the difficulty and extent of related surgery.

Various clinical evaluations have been performed for early PCF detection. Pharyngoesophagography (fistulography) is the most common radiologic evaluation for detection of PCF. This method can detect minor intramural defects and abnormal outpouchings and provide helpful dynamic information about the neopharynx following total laryngectomy [10]. However, its diagnostic accuracy is debatable because of its low sensitivity, ranging from 26–50% [11,12].

Many laboratory findings can suggest PCF. Elevated WBC and CRP are typical of inflammation and infection at the surgical site. However, the specificity of a single inflammatory marker is low because it can also be elevated by underlying diseases and pneumonia.

Therefore, in actual clinical practice, PCF is diagnosed by considering various clinical factors, although there are no standard guidelines. In this study, we investigated various clinical data including postoperative fever, serial blood labs, and fistulography for early PCF detection. We also developed prediction models using various combinations of clinical data for early PCF detection after TL. We evaluated the diagnostic accuracy of each model to identify the prediction models with the highest diagnostic accuracy.

## 2. Materials and Methods

### 2.1. Study Population

We reviewed the medical records of patients who received TL between 2004 and 2021 at Samsung Medical Center, and the Institutional Review Board approved this retrospective study. We included 263 patients who received TL ± partial or total pharyngectomy or cervical esophagectomy for treatment of primary and salvage laryngeal, hypopharyngeal, or esophageal cancer. The patients were classified into a no fistula group (*n* = 177, 67.3%) and a fistula group (*n* = 86, 32.7%).

Demographics, including age and sex, were collected. Clinical data were collected, including hospital days, surgical extent (primary tumor and cervical lymph nodes), and reconstruction methods. Routine blood laboratory measurements, including CBC, CRP, ESR, and chemistry profiles, were performed on postoperative days 3 (POD 3) and 7 (POD 7) and on additional days if there were signs of other major postoperative complications. Fistulography using meglumine diatrizoate dye (Gastrografin, Bracco, Monroe Township, NJ, USA) was routinely performed on POD 7, and oral intake was started when there were no signs of fistula on fistulography and blood labs.

### 2.2. PCF Definition and Management

PCF was determined by clinical evidence of salivary leak to the tracheostoma or neck wound. Suspected PCFs were confirmed by inspection of subcutaneous inflammation, saliva leakage, endoscopic examination of the fistula tract, surgical exploration, or definite contrast leakage on fistulography. When minor PCF was identified, management was conservative, including antibiotics, local wound dressing, negative pressure assisted dressing, and non-per-oral feeding with a nasogastric tube or gastrostomy tube. In cases of major fistula, which included diffuse necrosis, impending rupture of major vasculature, and no improvement or aggravation of PCF despite conservative management, we performed surgical interventions of wound debridement and/or flap reconstruction.

### 2.3. Clinical Factors Used for Early PCF Detection

The clinical factors collected for PCF detection were presence of fever (>38.0 °C) by POD 3, blood labs on POD 3 and POD 7, and fistulography at POD 7. Peripheral blood samples were collected using EDTA-anticoagulated vacutainer tubes. Complete cell counts, including white blood cells (WBC, ×10^3^/µL), Hb (g/dL), segmented neutrophils (%), and lymphocytes (%), were processed within 4 h of collection on the Sysmex XE2100 automated hematology analyzer (Sysmex Corporation, Kobe, Japan). Serum biochemical markers of C-reactive protein (CRP, mg/dL) and albumin (g/dL) were measured with the serum trace element concentrations by a Roche cobas 8000 c702 analyzer (Roche Diagnostics Corp., Indianapolis, IN, USA) as per the manufacturer’s instructions. Neutrophils-to-lymphocytes ratio (NLR) and CRP-to-albumin ratio (CAR) were analyzed. The ratios of POD 7 to POD 3 blood test values were calculated and compared between the no fistula and fistula groups.

### 2.4. Statistical Analysis to Compare the Clinical Factors between the Fistula and the No Fistula Groups

We compared clinical factors between the no fistula group (*n* = 177, 67.3%) and the fistula group (*n* = 86, 32.7%). The Mann–Whitney U test was used to compare continuous variables, and Fisher’s exact test was employed to analyze categorical variables between the two groups. *p*-value < 0.05 was considered statistically significant. Statistical analyses were performed using SPSS for Windows ver. 25.0 (SPSS Inc., Chicago, IL, USA).

### 2.5. Development of PCF Prediction Models

Because of the low diagnostic accuracy of fistulography alone, we combined clinical factors with fistulography and established scoring models for detecting PCF using various combinations. For selection of clinical variables for PCF detection, we introduced random forest and extreme gradient boosting machine-learning algorithms. The models were trained and validated using 10-fold cross-validation (CV) and bootstrap methods to calculate variable importance and feature selection. Clinical factors with high priority on machine-learning models were selected, and multiple logistic regression analyses using these clinical factors were performed to build scoring models.

After developing scoring models, the study subjects were randomly divided into a ratio of 3:1 for the holdout test. The scoring model trained in the former group was tested in the latter. The area under the receiver operating characteristic curve was used to evaluate the prediction performance of each model. The sensitivity and specificity were calculated using the cutoff point of each scoring model with Youden’s method. R 4.1.3 (R Foundation for Statistical Computing, Vienna, Austria. URL https://www.R-project.org/ accessed on June 2022.) was used for statistical analyses.

## 3. Results

A total of 263 patients underwent TL during the study period, and 82 (32.7%) developed PCF (Table 1). Mean age was not significantly different (*p* = 0.077) between the no fistula (64.9 ± 9.5 years) and fistula (64.6 ± 10.4 years) groups. The male-to-female ratio was not significantly different (*p* = 0.842) between the no fistula (156 and 21 patients, 88.1% and 11.9%, respectively) and fistula groups (75 and 11 patients, 87.2% and 12.8%). Hospital days were significantly fewer (*p* < 0.001) in the no fistula group (22.4 ± 23.8 days) than in the fistula group (55.9 ± 45.1 days). Primary surgery extent consisted of total laryngectomy (144 patients, 81.4%) and total laryngectomy with pharyngoesophagectomy (33 patients, 18.6%) in the no fistula group and total laryngectomy (65 patients, 75.6%) and total laryngectomy with pharyngoesophagectomy (21 patients, 24.4%) in the fistula group. Neck lymphatic surgery and flap reconstruction were performed in 47 patients (26.6%) and 42 patients (23.7%) in the no fistula group, respectively, and 17 patients (19.8%) and 25 patients (29.1%) in the fistula group, with no significant differences between the groups (*p* = 0.284 and *p* = 0.368). Previous treatment history was not significantly different (*p* = 0.527) between the fistula and no fistula groups. Previous treatment of the no fistula groups consisted of none (*n* = 80, 45.2%), radiation (*n* = 49, 27.6%), and chemoradiation (*n* = 48, 27.2%), and that of the fistula groups consisted of none (*n* = 37, 43.1%), radiation (*n* = 20, 23.2%), and chemoradiation (*n* = 29, 33.7%).

Postoperative clinical data were compared between the no fistula and fistula groups (Table 2). Fever (>38.0 °C) on POD 3 was more common (*p* < 0.001) in the fistula group (34.9%) than in the no fistula group (12.4%). For blood labs measured at POD 3, WBC, CRP, and NLR were significantly higher in the fistula group than in the no fistula group. In contrast, albumin and lymphocytes were significantly higher in the no fistula group than in the fistula group. CAR, Hb, and neutrophils were not significantly different between the two groups. For blood labs measured at POD 7, WBC, CRP, CAR, neutrophils, and NLR were significantly higher in the fistula group than in the no fistula group.

For the ratios of blood test values measured on POD 7 and POD 3, WBC, CRP, CAR, neutrophils, and NLR were significantly higher in the fistula group. Meanwhile, lymphocytes were considerably higher in the no fistula group. Leakage on fistulography was more common in the fistula group (29 patients, 38.2%) than in the no fistula group (5 patients, 3.0%). Fistulography, the most common detection method for PCF, showed a sensitivity of 33.7% and specificity of 97.2%, with an AUC of 0.675 (*p* < 0.001), suggesting that evaluating and managing PCF should not be determined with fistulography alone. Therefore, we tried to increase the predictive accuracy of PCF occurrence by adding measures to fistulography.

**Figure 1:**
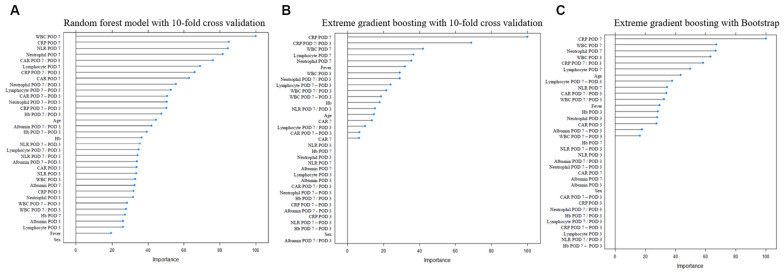
Machine-learning feature selection for development of prediction models for pharyngeocutaneous fistula (**A**) Random forest model with 10-fold cross validation. (**B**) Extreme gradient boosting with 10-fold cross validation. (**C**) Extreme gradient boosting with bootstrap.

We used machine-learning methods to find the appropriate variables. Feature selection using machine learning revealed the relative importance of each clinical factor (Figure 1).

We identified 10 major variables with reliable significance: WBC-POD7, NLR-POD7, CRP-POD7, CRP ratio, CRP difference (difference between POD7 and POD3), neutrophil ratio, neutrophil difference, CAR ratio, neutrophils-POD7, and CAR-POD7. By combining clinical factors with fistulography, we established multiple scoring models for PCF detection and selected the prediction models with the highest diagnostic performance.

**Model 1** probability of PCF = 1/(1 + exp(−2.573 + 2.893 * Fistulography + 1.145 * Neutrophil POD7 (65~75) + 1.659 * Neutrophil(75~85) + 2.853 * Neutrophil1(85<))^(−1))

**Model 2** probability of PCF = 1/(1 + exp(−8.6332 + 2.9405 * Fistulography + 1.6286 * log WBC POD7 + 4.7374 * Neutrophil ratio)^(−1))

Of the selected models, 1 and 2 showed the highest area under the receiver operating characteristic curve of 10-fold cross-validation, bootstrapping, and hold-out test. The diagnostic performance, including Sn, Sp, positive predictive value (PPV), and negative predictive value (NPV), was highest in model 2 (Table 3). In comparison with model 0 that was developed with fistulography alone (Sn: 0.37, Sp: 0.97, PPV: 0.85 and NPV: 0.75), model 2 (Sn: 0.83, Sp: 0.73, PPV: 0.60, and NPV: 0.90) showed better diagnostic performance for PCF detection.

## 4. Discussion

PCF occurs in 3.3–65% of cases after TL. It is a critical complication that may lead to major vessel ruptures, surgical site infection and necrosis, delayed adjuvant therapy, higher medical expenses, and even death [1,2,3,4,5,6]. Early-detected PCF can be managed conservatively with local wound dressing, antibiotics, and non-oral alternative feeding. In delayed diagnoses, increased risk of major vessel rupture or wound infection and necrosis may require additional extensive surgical intervention, including free flaps or major vessel ligations [8,9]. Therefore, early PCF detection has remained a significant issue after TL.

Many previous studies about PCF after TL have analyzed preoperative risk factors including underlying diseases and previous radiation treatment history to predict the occurrence of PCF before surgery [4,5,6]. However, we established a prediction model for early detection of PCF using clinical examinations during hospitalization after surgery, not preoperative factors, which could apply to real clinical situations.

The diagnostic methods for PCF detection vary because of a lack of consensus. Postoperative fever within 48 h after surgery, pain, and emphysema on neck X-ray showed significant correlation with PCF development [10,13,14,15]. However, these are non-specific markers that might correlate to other common postoperative conditions, such as wound infection, atelectasis, or pneumonia.

Therefore, objective evaluations such as imaging techniques are used for PCF detection, and contrast-mediated video fluoroscopy, esophagography, and fistulography have been the most common methods [9,11,12,16,17,18,19,20]. Fistulography is a practical, non-invasive tool with high specificity ranging from 91–100%. However, it also has low sensitivity from 26–50% [11,12,19,20,21]. As in previous reports, fistulography alone showed lower diagnostic performance in our study, with an Sn of 0.37 and Sp of 0.97. In this context, we tried to improve the diagnostic accuracy of fistulography using additional clinical data.

Laboratory results are potentially useful predictors of PCF. Wound amylase concentration collected from neck drainage of surgical sites has been introduced as an easily measured significant predictor of PCF [22,23,24]. The drawback of this technique might be that wound amylase could be a consequential finding of PCF and not useful for early detection and initiation of timely intervention [25]. Serum inflammatory markers have also been inspected as early PCF clues. Bradykinin, which is expressed in tumor samples, was introduced as a possible predictor for postoperative PCF development [26]. CAR on POD 2 to 4 showed an independent correlation with PCF presence [27]. Additionally, neutrophils and NLR were suggested as predictive PCF markers [28,29,30,31]. Postoperative hemoglobin <12.2 g/dL and albumin <3.5 g/dL were also suggested as PCF predictors [32,33]. In our study, we analyzed blood labs including WBC, CRP, neutrophils, lymphocytes, CAR, NLR at POD 3 and POD 7, and changes in these blood labs between POD 3 and POD 7.

Of the various candidates for early PCF detection, no single method could provide accurate diagnosis. Therefore, we considered multiple combinations of clinical features for early PCF detection. First, we selected significant clinical factors using machine-learning techniques (random forest, extreme gradient boosting) with 10-fold cross-validation and bootstrap to prevent overfitting. We developed several prediction models for diagnosing PCF using selected clinical factors, including WBC-POD7, NLR-POD7, CRP-POD7, CRP ratio, CRP difference between POD 7 and POD 3, neutrophil ratio, neutrophil difference, CAR ratio, neutrophils-POD7, and CAR-POD7. Finally, we fitted a model (Model 2) using fistulography, WBC at POD 7, and neutrophil ratio (POD7:POD3), and it yielded a high Sn (83%) and Sp (73%), which was not previously achieved by a single diagnostic method. Additionally, the established model consists of WBC, neutrophils, and fistulography, which are common measurements after TL.

If fistulography is negative for PCF but positive in our prediction model, further evaluation with enhanced neck computed tomography or esophagogastroduodenoscopy could be considered to localize the fistula site. In addition, careful wound care with more frequent observation and serial blood labs and images could also be considered. Using these prediction models of combinations of fistulography and inflammatory markers, early diagnosis can result in early intervention before critical complications from PCF.

In significant PCF cases, we should consider extensive surgery including major vessel ligation and flap reconstruction. However, if early PCF detection is possible with prediction models, PCF could be addressed with local therapy without major surgery and fatal complications. Many non-surgical treatments have been tried for PCF to prevent further progression and devastating complications such as major vessel rupture. In several case series, negative-pressure wound therapy yielded a high success rate for healing PCF [34,35,36]. Additionally, botulinum toxin injection and scopolamine patches were used to decrease salivary flow [37,38,39]. Hyperbaric oxygen therapy could also be considered, and fibrin glue applied to the suture site could prevent saliva damage to the surgical wound [39,40]. These treatment methods could be applied effectively in cases of early-detected PCF by prediction models.

In conclusion, we developed improved prediction models for early PCF detection using a combination of fistulography and blood inflammatory markers. However, the study has several limitations, including a retrospective design and use of data from a single center without external validation. Nonetheless, verification and implementation of our method could prevent fatal complications from PCF after TL by early detection.

## Figures and Tables

**Table 1:** Baseline characteristics of enrolled patients (*N* = 263).

	No Fistula	Fistula	*p*-Value
Patient no. (%)	177 (67.3)	86 (32.7)	Total = 263
Sex (M:F, *n*, %)	156:21 (88.1:11.9)	75:11 (87.2:12.8)	0.842
Hospital days (mean ± SD)	22.4 ± 23.8	55.9 ± 45.1	<0.001
Age (mean ± SD)	64.9 ± 9.5	64.6 ± 10.4	0.077
Primary surgery extent (*n*, %)			0.329
Total laryngectomy	144 (81.4)	65 (75.6)	
Total laryngectomy with pharyngoesophagectomy	33 (18.6)	21 (24.4)	
Neck lymphatic surgery (*n*, %)			0.284
No neck dissection	130 (73.4)	69 (80.2)	
Neck dissection	47 (26.6)	17 (19.8)	
Flap (*n*, %)			0.368
No flap	135 (76.3)	61 (70.9)	
Flap reconstruction	42 (23.7)	25 (29.1)	
Previous radiation or chemoradiation therapy			0.527
None	80 (45.2)	37 (43.1)	
Radiation therapy	49 (27.6)	20 (23.2)	
Chemoradiation therapy	48 (27.2)	29 (33.7)	

**Table 2:** Comparison of clinical factors between the no fistula and fistula groups (*N* = 263).

	No Fistula	Fistula	*p*-Value
Fever >38.0 °C by POD 3 (*n*, %)	22 (12.4)	30 (34.9)	<0.001
Blood labs POD 3			
WBC (mean ± SD, ×10^3^/µL)	11.4 (4.2)	13.0 (4.7)	0.040
CRP (mean ± SD, mg/dL)	6.9 (5.3)	7.3 (5.3)	0.799
Albumin (mean ± SD, g/dL)	3.1 (0.5)	3.0 (0.5)	0.022
CAR (mean ± SD)	2.3 (2.0)	2.5 (1.8)	0.734
Hb (mean ± SD, g/dL)	10.9 (1.5)	10.5 (1.5)	0.028
Neutrophils (%)	85.3 (6.7)	86.9 (6.6)	0.023
Lymphocytes (%)	7.9 (4.9)	7.0 (4.9)	0.024
NLR (mean ±S D)	16.4 (12.5)	21.6 (18.1)	0.026
Blood labs POD 7			
WBC (mean ± SD, ×10^3^/µL)	7.2 (2.7)	9.7 (3.5)	<0.001
CRP (mean ± SD, mg/dL)	2.7 (2.8)	4.8 (4.0)	<0.001
Albumin (mean ± SD, g/dL)	3.4 (0.4)	3.3 (0.4)	0.051
CAR (mean ± SD)	0.8 (0.9)	1.5 (1.4)	<0.001
Hb (mean ± SD, g/dL)	11.0 (1.4)	10.8 (1.4)	0.082
Neutrophils (%)	71.7 (10.2)	79.7 (8.3)	<0.001
Lymphocytes (%)	16.6 (7.6)	11.0 (5.4)	<0.001
NLR (mean ± SD)	5.7 (3.6)	10.2 (10.4)	<0.001
Ratio of POD 7:POD 3 values			
WBC (mean ± SD, ×10^3^/µL)	0.7 (0.3)	0.9 (0.7)	0.001
CRP (mean ± SD, mg/dL)	0.6 (0.8)	1.0 (1.0)	<0.001
Albumin (mean ± SD, g/dL)	1.1 (0.2)	1.1 (0.2)	0.292
CAR (mean ± SD)	0.5 (0.7)	0.9 (1.0)	<0.001
Hb (mean ± SD, g/dL)	1.0 (0.1)	1.0 (0.2)	0.374
Neutrophils (%)	0.8 (0.1)	0.9 (0.1)	<0.001
Lymphocytes (%)	2.6 (1.6)	2.2 (1.5)	0.013
NLR (mean ± SD)	0.4 (0.3)	0.7 (0.6)	0.001
Leakage on fistulography (*n*, %) *	5 (3.0)	29 (38.2)	<0.001

POD = postoperative day; CAR = CRP-to-albumin ratio; NLR = neutrophils-to-lymphocytes ratio. * Patients who did not undergo fistulography were excluded from the calculations.

**Table 3:** Predictive performance of clinical models for prediction of pharyngocutaneous fistula after total laryngectomy.

	Model 0	Model 1	Model 2
Analyzed clinical factors	Fistulography,	Fistulography, neutrophils (POD7)	Fistulography, WBC (POD7), neutrophil ratio (POD7:POD3)
Performance of prediction (AUROC)			
Apparent performance on whole dataset	0.68	0.82	0.83
10-fold CV	0.69	0.81	0.84
Bootstrap	0.68	0.82	0.82
Hold-out test set	-	0.77	0.75
Diagnostic power of prediction models			
Sensitivity	0.37	0.52	0.83
Specificity	0.97	0.91	0.73
Positive predictive value	0.85	0.73	0.60
Negative predictive value	0.75	0.80	0.90

POD = postoperative days; NLR = neutrophils-to-lymphocytes ratio.

## Data Availability

Data is available at https://onedrive.live.com/edit.aspx?resid=5660406f69699f24!42967 (accessed on 7 January 2023).
